# Donor substrate recognition in the raffinose-bound E342A mutant of fructosyltransferase *Bacillus subtilis *levansucrase

**DOI:** 10.1186/1472-6807-8-16

**Published:** 2008-03-17

**Authors:** Guoyu Meng, Klaus Fütterer

**Affiliations:** 1 School of Biosciences, University of Birmingham, Birmingham, B15 2TT, UK; 2 Current address : School of Crystallography, Birkbeck College, and Institute of Structural Molecular Biology, UCL and Birkbeck College, Malet Street, London, WC1E 7HX, UK

## Abstract

**Background:**

Fructans – β-D-fructofuranosyl polymers with a sucrose starter unit – constitute a carbohydrate reservoir synthesised by a considerable number of bacteria and plant species. Biosynthesis of levan (αGlc(1–2)βFru [(2–6)βFru]_n_), an abundant form of bacterial fructan, is catalysed by levansucrase (sucrose:2,6-β-D-fructan-6-β-D-fructosyl transferase), utilizing sucrose as the sole substrate. Previously, we described the tertiary structure of *Bacillus subtilis *levansucrase in the ligand-free and sucrose-bound forms, establishing the mechanistic roles of three invariant carboxylate side chains, Asp86, Asp247 and Glu342, which are central to the double displacement reaction mechanism of fructosyl transfer. Still, the structural determinants of the fructosyl transfer reaction thus far have been only partially defined.

**Results:**

Here, we report high-resolution structures of three levansucrase point mutants, D86A, D247A, and E342A, and that of raffinose-bound levansucrase-E342A. The D86A and D247A substitutions have little effect on the active site geometry. In marked contrast, the E342A mutant reveals conformational flexibility of functionally relevant side chains in the vicinity of the general acid Glu342, including Arg360, a residue required for levan polymerisation. The raffinose-complex reveals a conserved mode of donor substrate binding, involving minimal contacts with the raffinose galactosyl unit, which protrudes out of the active site, and specificity-determining contacts essentially restricted to the sucrosyl moiety.

**Conclusion:**

The present structures, in conjunction with prior biochemical data, lead us to hypothesise that the conformational flexibility of Arg360 is linked to it forming a transient docking site for the fructosyl-acceptor substrate, through an interaction network involving nearby Glu340 and Asn242 at the rim of a central pocket forming the active site.

## Background

Oligo- and polyfructosyl-sucrose polymers, collectively known as fructans, are synthesized by a significant number of bacteria and an estimated 40,000 plant species [1] either replacing or supplementing starch as a carbohydrate reserve. Fructans are synthesised from sucrose as the sole substrate, sharing a single sucrose starter unit (αGlc(1–2)βFru) to which fructofuranose units can become attached at various positions of the fructosyl or glucosyl ring, resulting in highly branched or linear polymers in a species-dependent fashion [2]. The two prevailing forms of fructans are β (2 → 6)-linked levan (αGlc(1–2)βFru [(2–6)βFru]_n_) and β (2 → 1)-linked inulin (αGlc(1–2)βFru [(2-1)βFru]_n_), with the degree of polymerization of fructans varying between a few hundred and several thousands saccharide units. In plants, fructans are thought to contribute to drought and frost tolerance by preventing rupture of cell membranes [2], whereas in bacteria fructans are known to serve as food storage and to contribute to biofilm formation [3].

While fructan synthesis in plants involves at least two enzymes with different fructosyl-donor and – acceptor specificities, levan or inulin synthesis in bacteria requires only a single enzyme, with sucrose initially acting as both fructosyl donor and acceptor substrate. Levansucrase (sucrose:2,6-β-D-fructan-6-β-D-fructosyl transferase, E.C.2.4.1.10), encoded in *Bacillus subtilis *by the *sacB *gene, catalyses the fructosyl transfer reaction

sucrose + acceptor → glucose + fructosyl-acceptor

*In vitro*, levansucrase mediates invertase (hydrolase) or polymerase activity depending on the concentration of the fructosyl donor substrate: below 250 mM, sucrose is cleaved into glucose and fructose with water acting as fructosyl-acceptor, whereas above this concentration levan production occurs through successive transfer of fructosyl units from sucrose to the fructosyl 6'-hydroxyl (assuming β (2 → 6)-linkage) of the acceptor substrate [4]. Levansucrase belongs to family 68 of glycoside hydrolases (GH) according to the classification of carbohydrate-active enzymes (CAZY, [5,6]). While structurally and functionally diverse, glycoside hydrolases share the requirement of two juxtaposed acidic side chains, acting as proton donor (to the leaving group) and catalytic nucleophile or catalytic base, respectively. Hydrolysis of the glycosidic bond can result in either inversion or retention of the anomeric configuration in the substrate, corresponding to a single or double displacement reaction mechanism, respectively [7]. Kinetic studies of levansucrase established that sucrose hydrolysis follows a Ping-Pong kinetic reaction mechanism that retains the anomeric configuration and involves a covalently bound fructosyl-enzyme intermediate [8-10].

**Figure 1:**
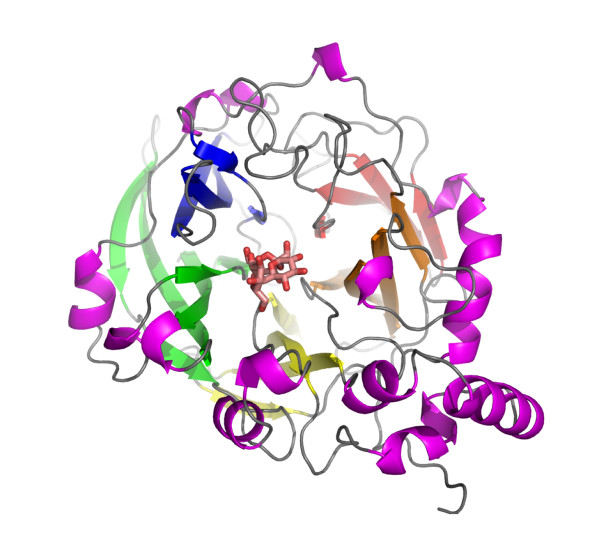
**The 5-bladed β-propeller fold of the catalytic domain of glycoside hydrolase families 68 and 32**. Ribbon diagram of *B. subtilis *levansucrase in complex with raffinose (shown as stick model).

We recently determined the crystal structures of *B. subtilis *levansucrase in the ligand-free form and bound to the fructosyl donor substrate sucrose [11]. Our structures established that the catalytic domain of GH family 68 enzymes folds into a 5-bladed β-propeller with the active site located in a deep axial pocket (Figure 1). This fold is shared by the catalytic domain of GH family 32 of retaining enzymes [12-15], as well as by the distantly related family 43 of inverting glycoside hydrolases [16,17] (see also reference [18] for a review of structure-function relationships in levansucrases). In agreement with a rich body of biochemical data [8,19-22], we proposed that the strictly conserved Asp86 (nucleophile) and Glu342 (proton donor) represent the two canonical catalytic carboxylate groups, while a third invariant carboxylate, Asp247, may aid catalysis by stabilizing the transition state of the oxocarbenium ion by forming close hydrogen bond contacts with two of the fructosyl hydroxyls [11]. While this assignment was confirmed in subsequent structural and biochemical studies of several ortho- and paralogs [12-15,23-25], the characterization of the structural determinants of levan synthesis has remained incomplete. In particular, the mode of and elements required for acceptor substrate binding remain unclear.

Here, we report crystal structures of three single-site mutants, D86A, D247A and E342A, previously shown to be catalytically inactive [11], in the ligand-free form, and that of the E342A mutant in complex with the fructosyl donor substrate raffinose. Comparisons between the ligand-free and substrate-bound structures shed light on a network of hydrogen bonds and ionic interactions surrounding the proton donor Glu342. This network reacts sensitively to presence or absence of the Glu342 carboxylate, and to changes in the ligand-binding state. We observed significant conformational flexibility of Arg360, a key residue in levan polymerisation, and propose that this role is linked to Arg360 alternating between alternative rotamer states, facilitating participation in a transient docking site for the fructosyl acceptor.

## Results

### *Apo *structures of inactive mutants D86A, D247A and E342A

**Figure 2:**
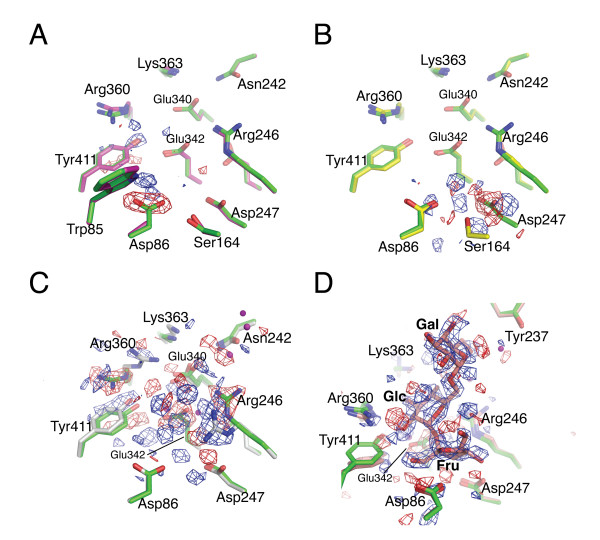
**Views of the active site of catalytically inactive mutants of *B. subtilis *levansucrase**. Difference electron density maps were calculated with Fourier coefficients (F_o,mutant _- F_o,wild-type_) and model phases derived from the wild-type enzyme structure (1OYG, [11]). The maps (2.1 Å resolution) are contoured at 5σ (panels **A**-**C**) or 4σ (panel **D**), with positive and negative density in blue and red, respectively. The stick models are colour-coded by carbon atoms as follows: wild type enzyme (green), D86A (magenta – panel **A)**, D247A (yellow – panel **B)**, E342A (grey – panel **C**) and raffinose-bound E342A (pale red – panel **D**).

Crystals of the inactive single site mutants D86A, D247A and E342A of *B. subtilis *levansucrase grew at similar solution conditions as the wild-type enzyme. Crystals were in space group *P*2_1_2_1_2_1_, diffracting to beyond 2.0 Å. Geometric constraints of the detector, rather than crystal quality limited data acquisition on the home source to a maximum resolution of 2.1 Å (Table 1). The diffraction data of mutant-forms of levansucrase were highly isomorphous to those of crystal form I of wild-type levansucrase (cf. [11]). Prior to structure (re-)building and refinement, structural differences were ascertained by way of difference Fourier maps (Figure 2), and the structures were refined starting from the wild-type model, altering the mutation sites to alanine (Table 2). The backbone structures superimpose closely with that of the wild-type enzyme. The root mean square deviation (RMSD) for 1760 backbone atoms between the mutant and wild type structures vary from 0.11 Å (D86A, E342A) to 0.12 Å (D247A). The RMSD for 1717 side chain atoms between mutants and wild type structures are 0.5 Å or less (0.33 Å – D86A, 0.36 Å – D247A, 0.48 Å – E342A), corresponding to ~2 times the estimated coordinate error at 2.1 Å. This indicates that overall the mutations induce only minimal structural changes. The missing carboxylate groups in the D86A and D247A mutants are clearly marked by negative density in the difference Fourier maps, but result in no other notable changes in the active site (Figures 2A and 2B).

**Table 1:** Crystallograhic data collection statistics

	D86A	D247A	E342A	E342A/Raffinose
Space group	*P*2_1_2_1_2_1_	*P*2_1_2_1_2_1_	*P*2_1_2_1_2_1_, 1	*P*2_1_2_1_2_1_
Unit cell				
*a *(Å)	51.1	51.2	51.1	52.2
*b *(Å)	67.1	67.4	67.3	66.7
*c *(Å)	123.7	123.8	123.6	124.0
Wavelength (Å)	1.5418	1.5418	1.5418	1.5418
Resolution range (Å)	30-2.07	30-2.07	30-2.07	30-2.03
Observations (*I*/*σ*(*I*) > 0)	80868	232052	118645	139045
Unique reflections (*I*/*σ*(*I*) > 0)	24114	26627	26368	27688
Last shell (Å)	2.14-2.07	2.14-2.07	2.14-2.07	2.10-2.03
*R*_sym _(%)^a,b^	3.9 (6.4)	3.8 (6.8)	3.7 (6.8)	5.3 (9.7)
<*I*/*σ*(*I*)>	24.7 (25.0)	25.0 (25.1)	25.0 (25.1)	25.1 (25.5)
Completeness (%)	90.8 (88.1)	99.5 (99.0)	98.8 (98.8)	98.2 (98.1)
Redundancy	3.4 (3.3)	8.7 (8.1)	4.5 (4.3)	5.0 (4.5)

^a^*R*_sym _= Σ(*I*-<*I*>)/Σ*I*

^b^high resolution shell in parentheses

**Table 2:** Statistics of crystallographic structure refinement

	D86A	D247A	E342A	E342A/Raffinose
Resolution range (Å)	30 – 2.1	30 – 2.1	30 – 2.1	30 – 2.1
*R*-factor (%)	16.2	16.8	18.0	17.9
*R*_free _(%)^a^	20.8	21.4	22.2	22.4
Total number of non-hydrogen atoms	3705	3711	3704	3748
Protein atoms	3439	3439	3438	3438
Water molecules	265	271	265	275
Ion sites	1	1	1	1
Raffinose molecules	0	0	0	1
RMSD from ideal values				
Bond length (Å)	0.009	0.009	0.009	0.007
Bond angle (°)	1.20	1.19	1.21	1.07
Main chain *B*-factors (Å^2^)	0.34	0.34	0.34	0.25
Side chain *B*-factors (Å^2^)	1.12	1.13	1.16	0.72
Wilson *B*-factor (Å^2^)	14.7	14.7	14.6	17.7
Average *B*-factor protein atoms (Å^2^)	14.1	13.4	15.2	15.4
Average *B*-factor solvent atoms (Å^2^)	19.3	19.6	21.9	16.7
Aver. *B*-factor (Å^2^) of raffinose	-	-	-	13.5
Ramachandran statistics^b^				
Most favoured regions (%)	88.5	89.3	88.3	88.8
Additionally allowed regions (%)	9.9	9.4	10.4	10.2
Generously allowed regions (%)	0.8	0.5	0.3	0.3
Disallowed regions (%)	0.8	0.8	1.0	0.8

^a^*R*_free _calculated using 5% of total reflections omitted from refinement

^b^Ramachandran statistics calculated using PROCHECK

In marked contrast, mutation of the general acid Glu342 to alanine has a profound impact on the rotamer state of functionally important side chains in the vicinity of the Glu342 carboxylate (Figure 2C). In the *apo *structure of wild-type levansucrase, Glu342 forms a tight salt bridge interaction with the guanido group of Arg246 (2.93 Å) and a strong hydrogen bond with the side chain hydroxyl of Tyr411 (2.67 Å) (Figure 2C). In E342A, the missing carboxylate prompts, firstly, the guanido group of Arg246, which is important for activity [24], to swing about 90° (about the Cγ-Cδbond) towards the axis of the β-propeller, overlapping in this configuration with the fructosyl binding site. Secondly, the E342A mutation eliminates the hydrogen bond to the Tyr411 hydroxyl, causing a minor upward shift of the phenol ring (~8°). Thirdly, Arg360 assumes an alternative rotamer state, involving an 80° rotation in χ_4 _and a near 90° rotation in χ_3_. This drastic change in rotamer configuration of Arg360, which is required for polymerase activity [20,22], is somewhat surprising: in wild-type levansucrase Arg360 interacts through a 2.7-Å hydrogen bond with Tyr411, but forms only a weak interaction with Glu342 (4.8 Å). The alternative rotamer state of Arg360 is ostensibly stabilised by a tight ionic interaction with Glu340 (2.9 Å), a residues involved in donor substrate binding (see [11] and below).

**Figure 3:**
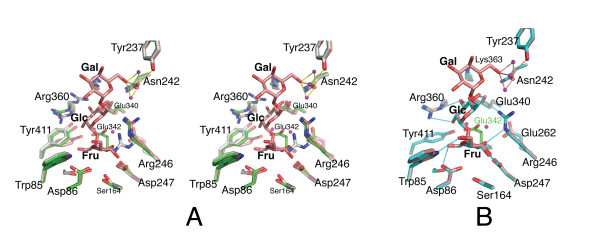
**Structural comparison of raffinose- and sucrose-bound E342A with *apo *wild-type and E342A levansucrase**. (**A**) Stereo diagram of the superimposition of *apo *wild-type (green), E342A (grey) and raffinose-bound E342A (pale red). (**B**) Superposition of sucrose-bound (1PT2, [11], light blue) and raffinose-bound E342A (this study). Dashed lines in cyan indicate H-bond interactions conserved between both complexes, those in red indicate additional contacts made by the galactosyl moiety.

In conclusion, the D247A and D86A mutations have little or no impact on the side chain configurations and interactions elsewhere in the active site, while the Glu342A substitution has knock-on effects for the network of non-covalent interactions around the Glu342 carboxylate. We note however, that binding of the donor substrate to levansucrase-E342A largely restores the side chain configuration of *apo *wild-type levansucrase (Figures 2D and 3A), prompting Arg246 and Arg360 to swing back into their original position.

### The raffinose-bound complex of levansucrase-E342A

Besides sucrose, levansucrase also accepts the trisaccharide D-raffinose (αGal(1-6)αGlc(1-2)βFru) as fructosyl donor. The products resulting from levansucrase-catalysed hydrolysis of raffinose are melibiose (Gal-Glc) and free fructose. Crystals of levansucrase-E342A were soaked in raffinose, followed by cryoprotection and flash-freezing in a 100 K nitrogen gas stream (see Methods). Diffraction data to 2.1 Å resolution were recorded in-house and difference electron density maps, comparing amplitudes of the raffinose-bound mutant and apo wild-type levansucrase, showed density that could be attributed unequivocally to the ligand (Figure 2D). With amplitudes on an (approximately) absolute scale, the occupancy of raffinose refined to a value near 1.0 (using CNS [26]), suggesting full occupancy, while the average B factor of the raffinose atoms was 13.5 Å^2 ^compared to 15.4 Å^2 ^for the protein atoms (Table 2). In the refined structure, the raffinose ligand displays a configuration in which the planes of the three sugar rings are approximately orthogonal to each other: the fructose moiety lies almost 'flat' on the bottom of the active site, the glucose moiety rises up and the galactose ring is jutting out into the solvent (Figures 2D and 3A). The fructose and glucose rings superimpose closely with their counterparts in the sucrose-bound complex [11] (Figure 3B), as illustrated by an RMSD of 0.26 Å for the atoms common between the two ligands.

**Figure 4:**
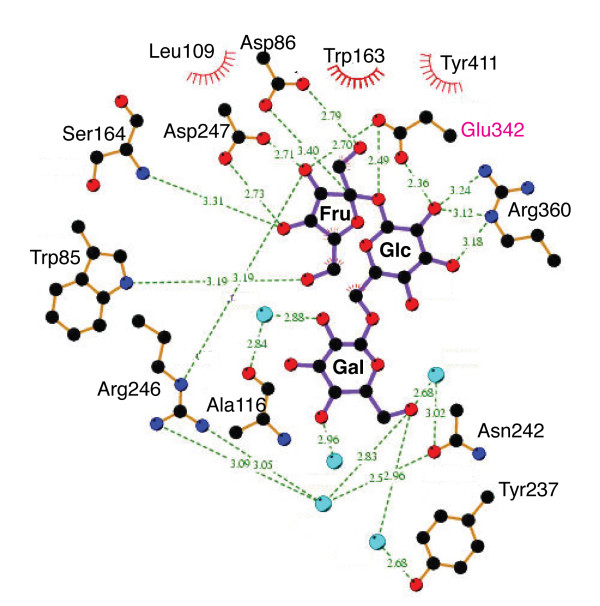
**Schematic diagram of interactions between raffinose and levansucrase E342A**. Interactions were calculated using LIGPLOT [41]. H-bond interactions, with distances in Å units, are indicated by dashed green lines. Residues making van der Waals or hydrophobic contacts are indicated by the 'bent comb' symbol. Water molecules appear as spheres in light blue, carbon, oxygen and nitrogen are in black, red and dark blue, respectively.

Following the terminology defined by Davies *et al. *[27], the active site of glycoside hydrolases can be divided into subsites with respect to the cleaved glycosidic bond. Applied to the present raffinose complex, subsite -1 coincides with the fructose, and subsites +1 and +2 with the glucose and galactose moieties, respectively (Figure 3). It is apparent that, with the exception of Arg246 and perhaps Glu342, side chains tend to form specificity-determining contacts with only one of the three subsites (Figures 3B and 4): the fructosyl moiety makes specificity-determining contacts with the side chains of Trp85 (3.1 Å to O6'), Asp86 (2.7 Å to O1'), Arg246 (3.2 Å to O3'), Asp247 (2.6 Å to O3' and 2.7 Å to O4'), and Glu342 (2.4 Å to O2' – assuming that the side chain conformation of Glu342 in the substrate-bound wild-type enzyme is identical to that of the ligand-free form). In subsite +1, the 2-, 3- and 4-hydroxyls of the glucosyl moiety form tight H-bond contacts (2.6 – 3.1 Å) with Arg360 and Glu340. These specificity-determining contacts lock the fructosyl and glucosyl units into defined orientations, positioning the anomeric carbon of the fructosyl unit within 3.2 Å of the nucleophile Asp86, and the glycosidic oxygen in close proximity (~2.5 Å) to the carboxylate of Glu342. In contrast, the galactosyl unit makes only few, water-mediated H-bonds, limited to the 6"-hydroxyl, with Asn242 and Tyr237, whereas the 2"-, 3"- and 4"-hydroxyl groups point into solvent (Figures 3A and 4).

The configuration of active site residues in raffinose-bound E342A is very similar to wild-type levansucrase in the ligand-free form, but contrasts with the *apo *form the E342A mutant. Owing to steric overlap with the fructosyl moiety, Arg246 swings back from the rotamer state in *apo*-E342A to the wild-type configuration. Similarly, Arg360 resumes the wild-type configuration, stabilised by H-bond interactions (~3.1 Å) with the 2-, 3-hydroxyls of the glucosyl ring. Yet, in the donor substrate-bound state Tyr411 tilts by ~20° towards the floor of the active site (Figures 2D and 3A). As a consequence, the H-bond between OH of Tyr411 and Nε of Arg360 is not preserved in either of the raffinose- and sucrose-bound complexes (Figure 3B). The dip of Tyr411 appears to result from van der Waals interactions with the glucosyl ring (3.4 Å) and with the guanido group of Arg360 (3.5 Å). Through the contacts to the ligand, Arg360 inserts deeper into the active site than in the *apo *structure of wild-type levansucrase. It remains unresolved whether the 20°-tilt of Tyr411 would also occur in a donor substrate complex of the wild-type enzyme (or a E342Q mutant).

It is noteworthy, that the position of the missing carboxylate of Glu342 is marked by two water molecules (Figure 3A), a feature consistent with the sucrose-bound complex [11]. Among the protein side chains, there is very little change between sucrose- and raffinose-bound structures of E342A (Figure 3B). The RMSD of side chain atoms located within 6.8 Å of the substrate (20 residues) is 0.26 Å. Thus within the limits of the estimated coordinate error the two structures are identical with respect to the protein framework.

## Discussion

**Figure 5:**
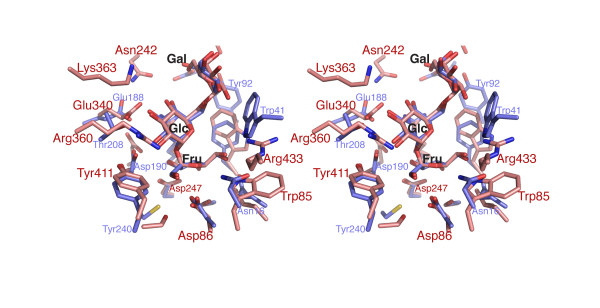
**Superimposition of raffinose-bound complexes of *T. maritima *invertase and *B. subtilis *levansucrase**. Raffinose-bound structures of *T. maritima *invertase (blue, 1W2T, [23]) and *B. subtilis *levansucrase-E342A (pale red) were superimposed by matching coordinates of three atoms of the raffinose ligand: fructosyl C2', C5' and glucosyl C5.

We present here the raffinose-bound complex of levansucrase-E342A in addition to the *apo *crystal structures of the three inactive point mutants D86A, D247A and E342A. Our previous study of the structure of *B. subtilis *levansucrase [11] established, based largely on structural arguments, the function of these three strictly conserved carboxylate side chains in the active site. The present raffinose complex reinforces a view that donor substrate recognition in *B. subtilis *levansucrase rests primarily on the common sucrosyl unit, whereas the galactosyl moiety, which protrudes out of the active site, makes only a few water-mediated H-bonds, pointing three unliganded hydroxyl groups to the bulk solvent. This mode of binding is echoed by the raffinose-bound complex of *Thermotoga maritima *invertase, which belongs to GH family 32 and which, like levansucrase, mediates hydrolysis of the glycosidic bond through a double displacement reaction mechanism [23]. In the latter study, an inert complex was facilitated by mutating the proton donor (Glu190) to aspartic acid. Superimposing the two complexes by matching the positions of 3 ligand atoms (Figure 5), reveals a very similar geometry of the ligand, and an almost perfect overlap of the catalytic residues. While there is significant variation of structural elements mediating specificity-determining contacts with the ligand, specific recognition of the outermost saccharide unit is weak in both structures and does not involve direct H-bonds. Nevertheless, the *T. maritima *complex includes notable van der Waals interactions between the galactose and Trp41, for which there is no counterpart in *B. subtilis *levansucrase (Figure 5). The importance of Glu340, Arg246 and Arg360 in forming specificity-determining contacts with the donor substrate is illustrated by the mutagenesis data obtained for levansucrase from *Bacillus megaterium *(74% identity on amino acid level). Mutating these side chains was reported as nearly abolishing hydrolase activity [24].

The functional assignment of the catalytic side chains in the active site of levansucrase raises the question of which structural features ensure that the requirement for differential protonation states of Asp86 (nucleophile) and Glu342 (general acid) [28] is met. Since the pH optimum of levansucrase lies in the range of pH 6.0 to 6.5 [4,24], the pKa of Glu342 must be raised to at least 6 – 6.5 in order to serve as the general acid. This could occur, for instance, through juxtaposition to hydrophobic or acidic side chains [29,30]. Yet, the shortest contacts of Glu342 (in the *apo *wild-type structure) are with Tyr411 (H-bond, 2.6 Å) and Arg246 (2.9 Å), neither of which is likely to result in the required effect. Still, a structure-based computational analysis using UHBD and scripts written by the Wade group [31-33] indicated a pKa of Glu342 at least 2 pH units above that of the free amino acid, arguing that a rise of the pKa is the result of the cumulative effect of the ensemble of side chains and contacts surrounding Glu342, an environment that includes three acidic side chains, Glu340 (4.1 Å), Glu262 (3.9 Å), and Asp247 (5.0 Å). Moreover, from the sucrose- and raffinose-bound complexes of the E342A mutant one can infer that donor substrate binding results in additional contacts with hydrophobic moieties of the sugar, and it is conceivable that a pKa shift only occurs upon substrate binding.

The distinct effects caused by the point mutation E342A on the configuration of adjacent side chains contrasts conspicuously with the minimal structural consequences of the D247A, D86A mutations. This is despite the fact that all three mutants crystallised on isomorphous lattices (Table 1). The conformational flexibility observed for Arg360 leads us to hypothesise that the interaction network, to which Glu342 is central, helps to coordinate donor and acceptor substrate binding.

Removal of the Glu342 carboxylate not only eliminates the H-bond to the Tyr411 hydroxyl (2.6 Å), but also weakens the H-bond between the Tyr411 hydroxyl and Nε of Arg360 (from 2.7 to 3.2 Å), as the latter assumes an alternative rotamer state, which is stabilised by the tight salt bridge interaction with Glu340 (2.9 Å). This observation suggests that, in the ligand-free state of wild-type levansucrase, the conformation of Arg360 is stabilized, firstly, through the H-bond to Tyr411, and, secondly, through the interaction with Glu342 (distance of 4.9 Å). This implies that in the ligand-free state (and at the condition of crystal growth, pH = 6.3) Glu342 may be deprotonated. It is conceivable that Arg360, in the absence of substrate, does not have a strong preference for the configuration seen in wild-type levansucrase, but may be free to assume the alternative conformation closer to Glu340, as both rotamer states of Arg360 occur with about the same frequency in protein structures (6.5% *vs*. 6%), and thus a switch between them is likely energy-neutral.

During the first reaction step of the double displacement mechanism, nucleophilic Asp86 forms a covalent intermediate with the fructofuranosyl, while Glu342 protonates the glucosyl leaving group [see panels A and B in Additional File 1]. Upon binding of the donor substrate (sucrose or raffinose), the Tyr411 phenol ring tilts towards the bottom of the active site, altering, and presumably weakening the interaction with Arg360. However, the conformation of Arg360 in the donor-bound state is stabilized by H-bonds (3.1 Å) to the 2-, 3-hydroxyls of the glucose moiety. Release of the leaving group deprives Arg360 of these stabilising contacts, and it may be free to switch to the alternative rotamer state, engaging in the salt bridge with Glu340, which also has lost its contacts to the substrate [panel B in Additional file 1].

In the second reaction step of the double displacement mechanism, the acceptor substrate binds [panel C in Additional file 1] and, through nucleophilic attack of the terminal 6'-hydroxyl (assuming a polymer with β (2 → 6) linkage) on the anomeric carbon, the enzyme-bound fructosyl is added to the acceptor. Based on the structural requirements for catalysis and the geometry of the active site, one would predict that the terminal fructosyl of the acceptor substrate binds in a position that overlaps at least partially with the site of the glucosyl leaving group, such that the 6'-hydroxyl is positioned appropriately for activation by Glu342 (now acting as general base).

The precise mode of acceptor substrate binding is as yet unclear. Located at the rim of the active site pocket, Asn242 has very recently emerged as a structural element required for polymerase activity [24], in addition to Arg360 [20,22]. Mutation of Asn252 to aspartate in *B. megaterium *levansucrase (corresponding to Asn242 in *B. subtilis*) preserves polymerase activity, but removal of the side chain amide (N252A, N252G) abrogates polysaccharide synthesis without affecting hydrolysis activity [24]. Accordingly, Homann *et al. *suggested that Asn252/Asn242 contributes to the acceptor-substrate binding site, identifying Asn252/Asn242 as a part of the +2 subsite (relative to the positioning of the fructosyl donor) [see panels C and D in Additional file 1]. In the present structure of *apo*-E342A, Arg360 and Asn242 are linked indirectly through H-bond/ionic interactions to Glu340 (Figures 2C and 3A), suggesting that all three side chains may form part of the fructosyl-acceptor binding site. Thus, we envisage a scenario where Arg360 can alternate between two rotamer states, which contribute to the donor and acceptor substrate binding sites respectively. Given the variety of oligosaccharide products synthesised by *B. megaterium *levansucrase and levansucrases of other species it appears that acceptor binding occurs with low specificity [24]. A flexible conformation of Arg360, acting as sort of a 'fishing hook', could contribute to accommodating acceptors in different orientations relative to the enzyme-bound fructosyl unit.

Weak affinity of acceptor binding may also explain the donor substrate concentration-dependent switch between invertase (< 250 mM) and polymerase (> 250 mM) activity. Bearing in mind that the -1 subsite is occupied by the fructosyl-enzyme intermediate following the first step of the double displacement mechanism, the acceptor will find a binding surface that, compared to the deep central pocket of *apo *levansucrase, offers significantly less depth to bury solvent-accessible surface. The raffinose complex, furthermore, illustrates that direct interactions between substrate and enzyme are limited to the -1 and +1 subsites of the donor substrate complex. Saccharide units beyond the +1 subsite might find it difficult to make specificity-determining contacts. In our observation, an intact set of interactions at the -1 subsite seems to be a prerequisite for 'high' affinity binding of the donor: when soaking crystals of the inactive D247A and D86A mutants in 500 mM sucrose for 30 min, we observed binding to a secondary site at a crystal packing interface, but no ligand was detected in the active site (data not shown), whereas lower concentrations (150 mM) and shorter soaking times (10 min) were sufficient to obtain full occupancy complexes with E342A using the same approach. This argues that productive binding of the donor depends on an intact set of interactions between the enzyme and the fructosyl moiety, and that the specific interactions of the glucose moiety, while conferring specificity, are less critical for achieving high affinity binding. Thus, in order to promote polymerisation, the acceptor substrate, which initially is sucrose, must be present at sufficiently high concentration to lead to productive binding and levan polymerisation.

## Conclusion

The data presented here are consistent with a view that donor substrate recognition in sucrose- or raffinose-bound complexes of GH32 and GH68-family enzymes rests primarily on the sucrosyl unit, a view that is in agreement with the structure of raffinose-bound *T. maritima *invertase. The recent activity data obtained for point mutants of *B. megaterium *levansucrase in conjunction with our structural data provide clues for the acceptor substrate binding site, a site to which Asn242, Glu340 and Arg360 appear to contribute. The biochemical and structural data lend support to the hypothesis that the conformational flexibility of Arg360 may play the role of a switch between donor and acceptor substrate binding modes.

## Methods

### Site directed mutagenesis

The single site mutants (D86A, D247A and E342A) were generated using the QuikChange mutagenesis protocol (Stratagene). The forward primer for the mutant, purchased from MWG, were as follows (base mutations resulting in a change of amino acid are highlighted in bold, one silent mutation is underlined):

5'-CTTCTGCAAAAGGGCTGGACGTTTGGG**C**CAGCTGGC-3' (D86A)

5'-CCATACGCTGAGAG**C**TCCTCACTACGTAG-3' (D247A)

5'-CAGTAACAGATGAAATTG**C**ACGCGCGAACGTC-3' (E342A)

A pET-11c plasmid with an insert encoding wild type *Bacillus subtilis *levansucrase was used as template. The polymerase chain reaction (PCR) mixture was prepared as follows: 5 μl of 10× *Pfu *buffer (Stratagene), 50 ng template DNA, 125 ng forward and reverse primers, 1 μl of a 5 mM mixture of dATP, dTTP, dCTP and dGTP, with H_2_O added up to a 50 μl reaction volume. In order to initiate the PCR, 1 μl of 2.5 units/μl *Pfu*Turbo DNA polymerase (Stratagene) was added upon heating the reaction mixture to 95°C. 12 PCR cycles were used for D247A and E342A. In each cycle, the program was set as follows: 30 sec at 95°C, 1 min at 55°C and 15 min at 68°C. 20 cycles was used for D86A. The setting of the PCR program was the same as those for D247A and E342A except for the annealing temperature (T_M _= 75°C). After the PCR, 1 μl of 10 units/μl *Dpn*I was added to each reaction mixture and incubated at 37°C for 1 h in order to digest the parental DNA. The resulting DNA was desalted using a gel purification kit (Qiagen), prior to transformation into electro-competent cells of *E.coli *DH5α. LB agar plates with ampicillin (50 μg/ml) were used to select cells containing the mutant DNA. Candidate colonies were first subjected to by restriction enzyme digest, then verified by DNA sequencing (Lark Technology).

### Structure determination of D86A, D247A and E342A

The mutant forms of levansucrase were purified and crystallised as described for the wild-type enzyme in [11]. Mutant crystals were cryo-protected with 20% (v/v) ethylene glycol and a 50:50 paraffin:paratone-N oil mixture. Diffraction data to 2.1 _ resolution data of the *apo *forms of levansucrase D86A, D247A, E342A were recorded on a DIP2030b image plate detector (MacScience) mounted on a FR-951 rotating anode generator (Cu-Kα) (Bruker AXS BV). All diffraction data were reduced using DENZO/SCALEPACK ver 1.97.2 [34]. All mutants crystallised in crystal form I ((51 × 67 × 125 _^3 ^unit cell)) of the wild type levansucrase [11] with one molecule per crystallographic asymmetric unit. The mutant models were fitted manually into electron density maps (σ_A_-weighted 2mF_o_-DF_c_, mF_o _- DF_c _and F_o(mutant) _- F_o(wild type) _maps) using O [35]. CNS [26] and REFMAC5 [36] were used to refine the model. Initial B-factors were refined after applying TLS correction (1 TLS group, 21 parameters) [37]. The final models were of excellent stereochemistry, with 99.7% of residues in allowed regions of the Ramachandran plot (PROCHECK). Three residues, Lys285, Lys393, and Thr431 were in a disallowed region of the Ramachandran plot, but their backbone conformation was confirmed in simulated annealing omit maps.

### Structure determination of the raffinose-bound E342A mutant

Crystals of E342A were soaked for 10 min in 150 mM raffinose plus mother liquor, then cryo-protected in 20% (v/v) ethylene glycol containing 150 mM raffinose. Residual mother liquor was removed by briefly immersing the crystal in a paraffin:Paratone-N oil mixture. X-ray diffraction data of the raffinose-bound mutant E342A were recorded in-house and processed as above. A model of D-raffinose (αGal(1-6)αGlc(1-2)βFru) was generated using SYBYL (Tripos Inc.), and dictionary files (*e.g. *torsion file for program O and parameter file for CNS) were obtained using MOLEMAN2 [38] as implemented at the Hic-up server [39]. The raffinose model was fitted manually into the difference electron density maps calculated using phases of the refined wild type model and difference amplitudes [F_o(raffinose: E342A) _- F_o(wild type)_] (Figure 2D). The fitting procedures were carried out using O [35]. The substrate bound models were refined using REFMAC5 [36] with two TLS groups, corrsponding to protein and substrate, respectively.

### Coordinates

Coordinates and structure factors have been deposited at the Protein Data Bank [40] under the accession codes: [PDB:3BYJ] [PDB:3BYK] [PDB:3BYL] [PDB:3BYN] describing the structures of mutants D86A, D247A, E342A and raffinose-bound E342A, respectively.

## Authors' contributions

GM and KF designed the study. GM generated reagents, produced proteins, crystals and performed the crystallographic analysis. GM and KF designed figures and wrote the manuscript. All authors have read and approved the manuscript.

## Supplementary Material

Additional file 1 **Schematic of levan polymerisation**. Schematic diagram of the model of the reaction cycle of levansucrase-catalysed fructosyl polymerisation as proposed in the main text. Selected non-covalent interactions are indicated by dashed lines. Numbers in bold indicate the subsites with respect to the cleaved glycosidic bondin, as adopted from reference [27]. (**A**) The initial complex of the fructosyl donor substrate; the nucleophile Asp86 is deprotonated while the general acid Glu342 is in the protonated state. (**B**) Following hydrolysis of the glycosidic bond, the glucose moiety is released and the fructosyl is covalently bound to the nucleophile; Arg360 assumes the alternative rotamer state and forms an ionic interaction with Glu340. (**C**) Binding of the acceptor substrate (here, a second sucrose molecule), mediated by Arg360 and Asn242; Glu342 is deprotonated and ready to activate the terminal hydroxyl of the acceptor for nucleophilic attack onto the enzyme-bound fructosyl. (**D**) Release of the elongated acceptor; Arg360 returns to the original conformation. Click here for file
