# Supplementary figures and images for: Donor substrate recognition in the raffinose-bound E342A mutant of fructosyltransferase Bacillus subtilis levansucrase
Source: BMC Struct Biol. 2008 Mar 17;8:16. doi: 10.1186/1472-6807-8-16 (PMC2277421; doi:10.1186/1472-6807-8-16)

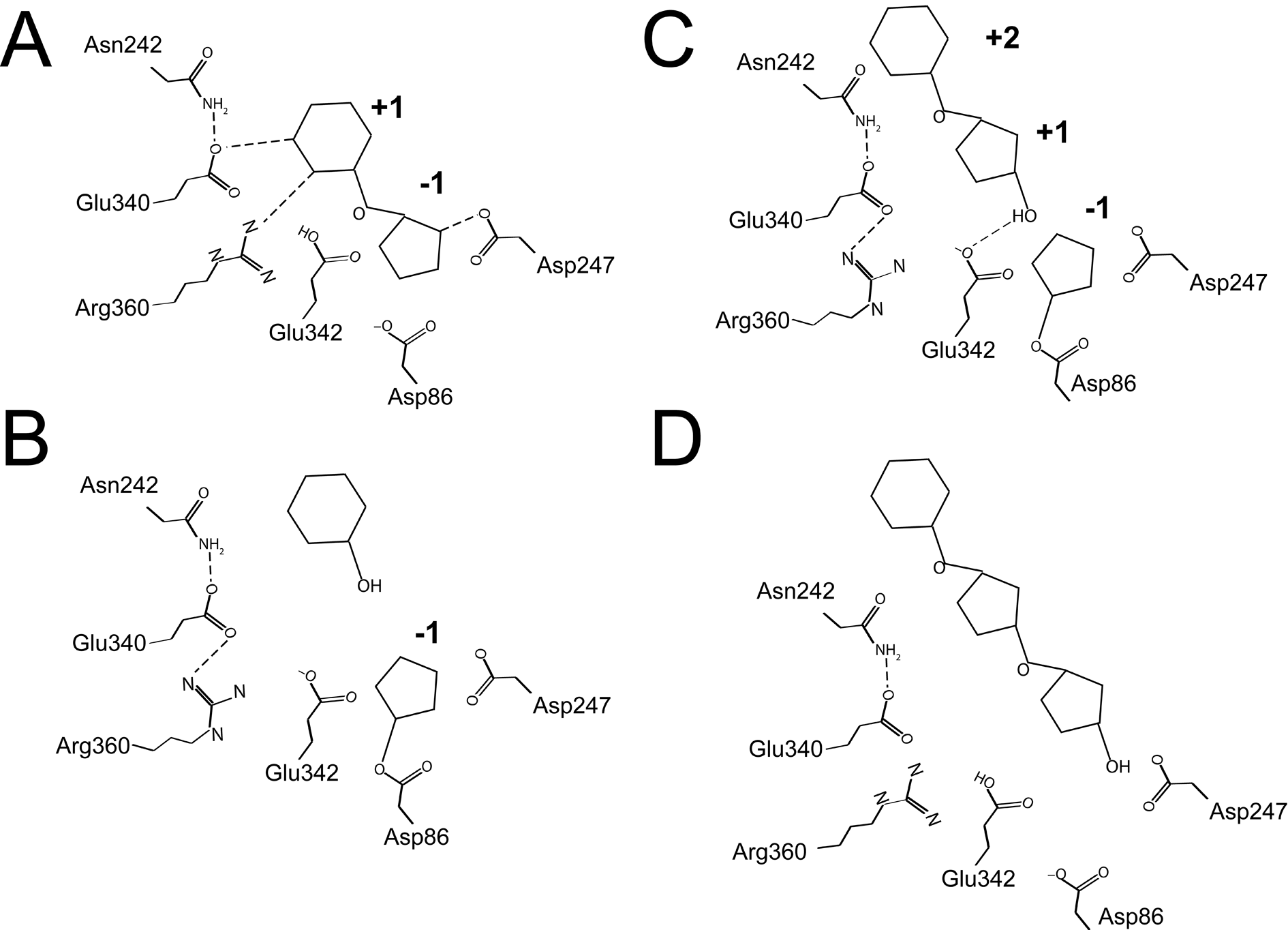

Supplement: Additional file 1 — Schematic of levan polymerisation. Schematic diagram of the model of the reaction cycle of levansucrase-catalysed fructosyl polymerisation as proposed in the main text. Selected non-covalent interactions are indicated by dashed lines. Numbers in bold indicate the subsites with respect to the cleaved glycosidic bondin, as adopted from reference [27]. (A) The initial complex of the fructosyl donor substrate; the nucleophile Asp86 is deprotonated while the general acid Glu342 is in the protonated state. (B) Following hydrolysis of the glycosidic bond, the glucose moiety is released and the fructosyl is covalently bound to the nucleophile; Arg360 assumes the alternative rotamer state and forms an ionic interaction with Glu340. (C) Binding of the acceptor substrate (here, a second sucrose molecule), mediated by Arg360 and Asn242; Glu342 is deprotonated and ready to activate the terminal hydroxyl of the acceptor for nucleophilic attack onto the enzyme-bound fructosyl. (D) Release of the elongated acceptor; Arg360 returns to the original conformation. [file 1472-6807-8-16-S1.png]
